# Supercritical Extraction and Compound Profiling of Diverse Edible Mushroom Species

**DOI:** 10.3390/foods14010107

**Published:** 2025-01-02

**Authors:** Slađana Krivošija, Nataša Nastić, Milica Karadžić Banjac, Strahinja Kovačević, Sanja Podunavac-Kuzmanović, Senka Vidović

**Affiliations:** Faculty of Technology Novi Sad, University of Novi Sad, Boulevard cara Lazara 1, 21000 Novi Sad, Serbia; sladjana.krivosija@uns.ac.rs (S.K.); natasa.nastic@uns.ac.rs (N.N.); mkaradza@uns.ac.rs (M.K.B.); strahko@uns.ac.rs (S.K.); sanya.podunavac@uns.ac.rs (S.P.-K.)

**Keywords:** edible mushrooms, supercritical carbon dioxide extraction, sterols, fatty acids, chemometric analysis

## Abstract

Mushrooms are a raw material rich in many nutritional compounds, and that is why a number of them are widely known as functional food. They contain fatty acids, carbohydrates, lycopene, sterols, lovastatin, trace elements, and other valuable compounds that show a wide range of properties, such as hepatoprotective, anticancer, antiviral, etc. For more efficient utilisation of mushrooms’ biologically active substances, widespread supercritical carbon dioxide extraction (Sc-CO_2_) was used as an efficient way to isolate the high-value phytoconstituents from this type of raw material. Using Sc-CO_2_, the extracts of five types of edible mushrooms—*Lycoperdon saccatum*, *Pleurotus ostreatus*, *Craterellus cornucopioides*, *Russula Cyanoxantha* and *Cantharellus cibarius*—were obtained. During the Sc-CO_2_ process, the extraction time was reduced to 4 h compared to the prolonged process time applied in the typical traditional techniques (6–24 h). The extraction pressure (30 MPa) and temperature (40 °C) were constant. Fatty acids and the compounds of steroid structures were determined in the obtained extracts using GC–MS and GC–FID methods of analysis. The dominant compounds identified in the lipid extracts were fatty acids (linoleic, oleic, palmitic and stearic) and sterols (ergosterol, 7,22-ergostadienone and 7,22-ergostadienol). For complete insight into the process and to obtain the value of the extracts, chemometric analysis is provided. Principal component analysis (PCA) and hierarchical cluster analysis (HCA), as well as k-means clustering, showed that *Craterellus cornucopioides* was distinguished based on the extraction yield results.

## 1. Introduction

Due to the significant presence of nutritional components, mushrooms are well known as a valuable functional food. However, despite this knowledge, mushrooms are one of the main underutilised sources of nutritious food. According to El Sheikha and Hu [1], of the 14,000 reported species of mushrooms, 3000 are recognised as edible, and among them, 270 species are known to have therapeutic potential that can enhance human health. Their nutritional importance is reflected in the presence of a high amount of fibre and nutrients such as proteins, vitamins and minerals, along with a low content of fat and calories [2]. According to Rathore et al. [3], their composition accounts for 50–65% carbohydrates, 19–35% proteins and a small amount of fat (2–6%). When discussing the nutritional composition, carbohydrates should be singled out as one of the main ingredients in the dry matter of edible mushrooms. Both digestible (glycogen, mannitol, glucose, trehalose) and non-digestible (chitin, β-glucan and mannan) carbohydrates are present. Furthermore, mushrooms also contain polysaccharides, glycoproteins and various secondary metabolites (polyphenols, alkaloids, acids, sterols, etc.) [4]. Further, amino acids are present in mushrooms [5], and mushrooms’ raw material is rich in fatty acids, of which polyunsaturated fatty acids (PUFA) are more prevalent and in a higher proportion compared to saturated fatty acids [6]. From the nutritional side, mushrooms are noteworthy because they do not contain gluten or cholesterol and contain PUFA up to 75% of the total reported lipids [7]. Mushrooms represent a vegetative source of vitamin D, vitamin B and various minerals (iron, phosphorus, etc.) needed for human physiological functions [3,8,9].

Edible mushrooms are known for their characteristic texture, taste and, above all, medicinal properties. They can be used either directly in the diet or individual fractions obtained from them can be consumed to promote health [10,11]. Due to the presence of these highly valuable bioactive compounds, mushrooms are considered important because they can possess hepatoprotective, anticancer, antiviral, immune-enhancing and hypocholesterolemic properties [6]. Because of their low-fat and high fibre content, as well as the presence of natural antioxidants, their use in the prevention of cardiovascular diseases is very important [2]. Mushrooms are of significant scientific interest due to their therapeutic potential and a wide range of medicinal properties, including antitumor, antioxidant, anti-inflammatory, antimicrobial and cardiovascular, among others [12,13].

For obtaining active compounds from edible mushrooms, the efficient destruction of the cell wall to release intracellular material needs to be provided [14]. Traditional extraction methods that can be applied for the isolation of these compounds have several disadvantages, such as the high consumption of organic solvents (mostly n-hexane and methylene chloride), high processing temperature and a long extraction time (6–24 h, sometimes even longer). Due to this, conventional techniques are accompanied not only by high cost and low efficiency but also by a negative impact on the environment [15,16,17,18]. Also, using these types of extraction, it is possible to produce a negative effect in the form of decomposition and coagulation of active ingredients [19]. To mitigate or eliminate these disadvantages, advanced, attractive extraction methods such as ultrasound-assisted, subcritical water and supercritical carbon dioxide extraction (Sc-CO_2_) have been developed. These technologies are primarily highly efficient and environmentally friendly and show great potential for the production of extracts applicable in health care medicine and foods as active ingredients. Their mechanism of action usually refers to the treatment of cell damage by physical means to increase the extraction yield and improve product quality [20,21,22].

The extraction technology based on the use of Sc-CO_2_ appears as a modern, attractive method for isolating natural compounds with added value, i.e., as an alternative that includes the principles of “green chemistry”, which is inert, non-toxic, cheap and widely available, with a moderate critical pressure and temperature that successfully prevents the decomposition of thermolabile compounds [23]. By manipulating temperature and/or pressure, it is possible to adjust the properties of CO_2_, such as density, increased solvent selectivity and other physicochemical properties, which further results in different extraction yields and extract compositions [24]. Also, one of the leading advantages of this technology is obtaining extracts without solvents, which avoids the need for additional purification and thus greatly reduces costs. Additionally, material left after extraction is applicable for further ingredient isolation, as it is not contaminated by any solvent and therefore can be applied for further production of proteins, antioxidants, carbohydrates, etc.

Wild edible mushroom consumption is only seasonal and used by certain groups of populations (gourmets, enthusiasts and local people) [25]. This, as well as mushrooms’ short shelf life, are the main reasons why their bioactive constituents are underutilised [8]. Therefore, the aim of this study was to investigate the effectiveness of the application of Sc-CO_2_ extraction for the isolation of high-value compounds from five species of edible mushrooms, Pestle puffball (*Lycoperdon saccatum*), Oyster mushroom (*Pleurotus ostreatus*), Black trumpet (*Craterellus cornucopioides*), Variegated russula (*Russula Cyanoxantha*) and Golden chanterelle (*Cantharellus cibarius*). Sc-CO_2_ extraction has been selected as the method of choice for the processing of these five mushrooms, as it enables the efficient extraction of low-polar valuable constituents. And on the other hand, the material left after extraction can be processed further for the safe production of other ingredients of value. This further processing of material left after extraction is possible as Sc-CO_2_ extraction leaves no trace of any extraction solvent applied, and the thermal degradation or thermal transformation of the present constituents is avoided as processing is achieved at mild temperature conditions. This means that after low-polar fractions are separated from the mushroom, this material can be applied for the safe and efficient recovery of, e.g., proteins.

Taking into account the growing interest in edible mushroom application, interest in fatty acids for human and clinical nutrition, as well as interest in a new resource of certain sterolic bioactives such as ergosterol, we sought to produce and highlight the profile of the non-polar compounds present in the supercritical extracts and to estimate their potential further use. Using a pressure of 30 MPa, which through our previous research [26,27] proved to be the most effective during Sc-CO_2_ extraction, we evaluated the extraction efficiency based on the extraction yield; after which, gas chromatography–mass spectrometry (GC–MS) and gas chromatography–flame ionisation detector (GC–FID) analysis of the obtained lipid extracts was performed in order to identify the dominant compounds of the lipid fraction of the mushroom isolated in this way. Since chemometric analysis in the domain of supercritical extractions is currently successfully used [28,29], principal component analysis (PCA) and hierarchical cluster analysis (HCA) as pattern recognition chemometric methods were used in this paper for experimental data analysis and interpretation.

## 2. Materials and Methods

### 2.1. Plant Material and Chemicals

Mushrooms—Pestle puffball (*L. saccatum*), Oyster mushroom (*P. ostreatus*), Black trumpet (*C. cornucopioides*), Variegated russula (*R. Cyanoxantha*) and Golden chanterelle (*C. cibarius*)—were collected in the area of Istria, Croatia. Fresh mushrooms were air-dried and then stored in hermetically sealed plastic bags at room temperature. Before extraction, dried mushroom samples were ground in a blender. Using vibrating sieves (CISA, Cedaceria, Spain), the degree of fragmentation of the plant material was determined. The mean particle diameter was 0.132 mm, 0.277 mm, 0.433 mm, 0.294 mm and 0.347 mm, respectively.

For Sc-CO_2_, commercial CO_2_ (purity 99.9% *v*/*v*) was used (Messer, Novi Sad, Serbia). All other chemicals used during the experiment were analytical purity reagents.

### 2.2. Sc-CO_2_ Extraction

High-pressure extraction was performed using a laboratory high-pressure extraction plant (HPEP, NOVA-Swiss, Effretikon, Switzerland) with Sc-CO_2_ as the extraction solvent. The primary specifications of the Sc unit encompass the following components: a gas cylinder containing carbon dioxide (CO_2_), a diaphragm-type compressor with a pressure capacity extending up to 1000 bar, an extractor vessel equipped with a heating jacket for the heating medium (featuring an internal volume of 200 mL and a maximum operating pressure of 700 bar), a separator incorporating a cooling jacket for the cooling medium (with an internal volume of 200 mL and a maximum operating pressure of 250 bar), a pressure control valve, a temperature regulation system and regulation valves.

The extractions were performed at a pressure of 30 MPa, temperature of 40 °C and CO_2_ flow of 1.94 kg/h. Extraction yield (EY) was measured after 0.5, 1, 1.5, 2, 3 and 4 h of extraction, in order to study the kinetics of the process. The separator conditions were 1.5 MPa and 23 °C. Each extraction was carried out in triplicate. The obtained extracts were placed in glass bottles, sealed and stored at 4 °C to prevent any possible degradation until the analysis. The EY was expressed as the mass of collected extract (in grams) per gram of dry plant material, i.e., percentage (%).

### 2.3. Soxhlet Extraction

The ground plant material (5 g) was transferred to an extraction flask, which was placed in a *Soxhlet* apparatus with an attached reflux condenser. Hexane (150 mL) was then added to the flask and used as a solvent. After 8 h (480 min) of extraction and complete exhaustion of the mushroom material, the solvent was removed by evaporation on a rotary vacuum evaporator and the extraction yield was determined. Extraction was performed in triplicate.

### 2.4. GC–MS Analysis of Sc-CO_2_ Mushroom Extracts

For GC–MS analysis, the samples of the supercritical mushroom extracts (200 mg) were extracted with 1 mL of acetonitrile for 15 min in an ultrasonic bath. The acetonitrile extracts were then filtered over 50 µm filters and injected in the GC–MS system and Agilent 7890A GC system coupled to a quadrupole mass spectrometer model Agilent 5975C. For mushroom extract analysis, the GC was fitted with a capillary HP-5MS column with a 0.25 µm film thickness, 30 m length and inner diameter of 0.25 mm. The operating conditions were as follows: injector temperature (250 °C); split ratio (20:1); detector temperature (300 °C); carrier gas He with constant pressure of 21.956 psi. The temperature program was from 60 to 300 °C (3 °C/min). The mass spectrometry (MS) conditions were as follows: ionisation voltage (70 eV); ion source temperature (230 °C); scan range *m*/*z* (35–550). The acetonitrile extracts (2 µL) were injected. The identification of the individual compounds in mushroom supercritical extracts was based on computer matching with the Adams and NIST/EPA/NIH version 2.0d mass spectral libraries. The constituents of the extracts were also identified by comparing their retention times to those with the Adams library. For methyl ester analysis, the GC was fitted with a capillary DB-23 column with a 0.25 pm film thickness, 30 m length and inner diameter of 0.25 mm. The operating conditions were as follows: injector temperature (250 °C); split ratio (20:1); detector temperature (300 °C); carrier gas He with constant pressure of 37.7 psi. The temperature program was from 200 to 240 °C (5.6 °C/min) and held at 240 °C for 15 min. The mass spectrometry (MS) conditions were the same as those for intact extract analysis [30].

### 2.5. GC–FID Analysis

For GC–FID analysis, the supercritical mushroom extracts were transesterified. The extracts (200 mg) were dissolved in 2 mL of sulfuric acid (3%) in methanol. The vials with reaction mixtures were sealed and heated in a water bath for 2 h. After cooling, the mixture was neutralised with 2 mL of NaHCO_3_ solution, and 2 mL of CH_2_Cl_2_ was added. The organic layer was dried over anhydrous Na_2_SO_4_, filtered and evaporated under reduced pressure. The residue was dissolved in CH_2_Cl_2_ and injected into the GC system. For the GC–FID analysis of methyl esters, a Hewlett-Packard chromatograph model HP 5890 Series Il equipped with a FID was used and fitted with the same column as those for GC–MS analysis (DB-23). The operating conditions were as follows: injector temperature, 250 °C; split ratio, 30:1; detector temperature, 300 °C; carrier gas H_2_ with flow rate, 1.0 mL/min. The temperature program was from 150 °C to 240 °C (4 °C/min) and held at 240 °C for 10 min. An amount of 1 μL of the sample dissolved in CH_2_Cl_2_ (1:100 *v*/*v*) was injected. The percentage composition was computed by the normalisation method from the GC (FID) peak areas [30].

### 2.6. Chemometric Methods

Chemometric analysis was carried out on a data set consisting of extraction yield results of five types of edible mushrooms. Since experimentally obtained data were on the same scale, PCA and HCA were performed on the non-scaled data and were carried out using Statistica version 14.0.0.15 [31] and NCSS 2023 [32] software. As is well known, the PCA analysis results are presented using score and loading plots. Scores represent the new coordinates of the projected objects while loading reflects the direction with respect to the original variables. HCA analysis searches for objects that are close together in the variable space and groups them into clusters [33]. The clustering was performed on the basis of Ward’s method by calculating Euclidean distances. In order to discriminate the studied mushroom samples and to reveal similarities and dissimilarities among them, the clustering hierarchy was presented in the form of clustered heat maps (double dendrogram). The horizontal axis on the dendrogram represents the distance or dissimilarity between the clusters [34]. The heat maps enable clear insight into individual variables’ influence in the course of grouping in the clusters. Additionally, k-means clustering, as a machine learning algorithm, was used for grouping.

### 2.7. Statistical Methods

Statistical analysis for EYes during Sc-CO_2_ extraction and *Soxhlet* extraction was performed using one-way ANOVA statistical analysis, followed by Tukey’s test. Since each extraction was performed in triplicate, the results are expressed as mean value ± standard deviation (SD). Mean values were considered significantly different at the *p* < 0.05 confidence level.

## 3. Results

### 3.1. Sc-CO_2_ Extraction of Mushrooms

The results of Sc-CO_2_ extraction of the investigated five different types of edible mushrooms are given in Table 1. It can be clearly seen that the yield of all mushrooms increases with increasing time, and this yield is intense in the first three hours of extraction. A significantly lower yield compared to other species was achieved with *P. Ostreatus* (0.807 ± 0.61%), which means that this species is lower in the content of lipid components. The highest yield was given by *C. cornucopioides* (3.316 ± 0.05%), followed by *C. cibarius* (2.180 ± 0.48%), *R. Cyanoxantha* (2.061 ± 0.02%) and *L.saccatum* (1.664 ± 0.25%).

With the intention of proving the greater efficiency of this green, attractive extraction technique, a comparison with the traditional *Soxhlet* extraction was made for all analysed mushroom species, namely *L. saccatum*, *P. Ostreatus*, *C. cornucopioides*, *R. Cyanoxantha* and *C. cibarius*. The yield obtained by *Soxhlet* was 1.75%, 0.83%, 3.38%, 2.11% and 2.47%, respectively, for the mentioned types of mushrooms. However, the use of organic solvents, a long extraction time and obtaining extracts of unequal quality are just some of the disadvantages of this type of extraction. Therefore, such a small difference in yield should be considered negligible compared to all the benefits that Sc-CO_2_ extraction offers.

### 3.2. GC–MS and GC–FID Analysis of Sc-CO_2_ Mushroom Samples

In order to highlight the bioactive profile and to define the composition of fatty acids in the supercritical mushroom extracts, GC–MS and GC–FID analysis were performed. The basic compounds detected by GC–MS analysis were free carboxylic acids (mainly oleic and linoleic), as well as compounds of some other classes (aldehydes, alcohols, steroids, aromatic components). After transesterification, methyl esters of carboxylic acids were detected using GC–MS analysis and quantified using GC–FID analysis. Table 2 shows the compound profile identified by GC–MS.

GC–FID was further used to quantitatively assess the fatty acid composition of triglycerides in the supercritical mushroom extracts. Before analysis, transesterification was performed. The results obtained from this analysis are shown in Table 3.

### 3.3. Chemometric Analysis

**Figure 1:**
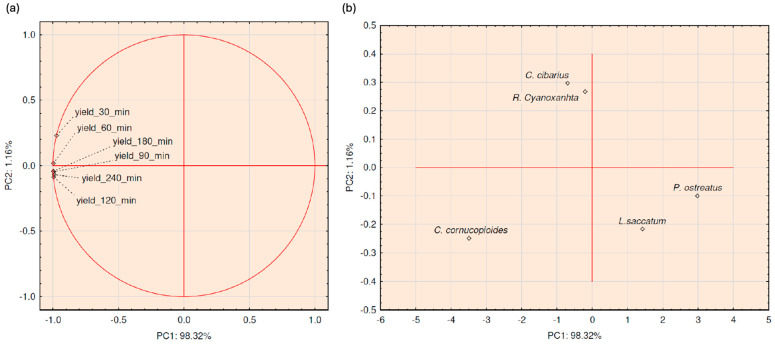
Score (**a**) and loading (**b**) plots of conducted PCA analysis covering extraction yield results.

In the first iteration, PCA was carried out using extraction yield data, as shown in Figure 1. PCA analysis gave the model described through two PCs, with a total variance of 98.32% for PC1 and 1.16% for PC2. The score plot (Figure 1a) indicated that along the PC1 axis for the mushroom samples’ positioning on the loadings plot, all six extraction yield results had the same influence, with negative coefficients of latent variables ranging from −0.9727 to 0.9976. From the loading plot (Figure 1b), it can be seen that *L. saccatum* and *P. ostreatus* were positioned in the cluster on the positive end of the PC1 axis. On the negative end of the PC1 axis, *C. cibarius* and *R. Cyanoxantha* were positioned in the cluster, while *C. cornucopioides* is out of the clusters. Observing the PC2 axis, the most dominant positive influence that had an extraction yield resulted at 30 min. On the positive end of the PC2 axis, *C. cibarius* and *R. Cyanoxantha* were placed, while *L. saccatum* and *P. ostreatus* were placed on its negative end together with *C. cornucopioides*.

**Figure 2:**
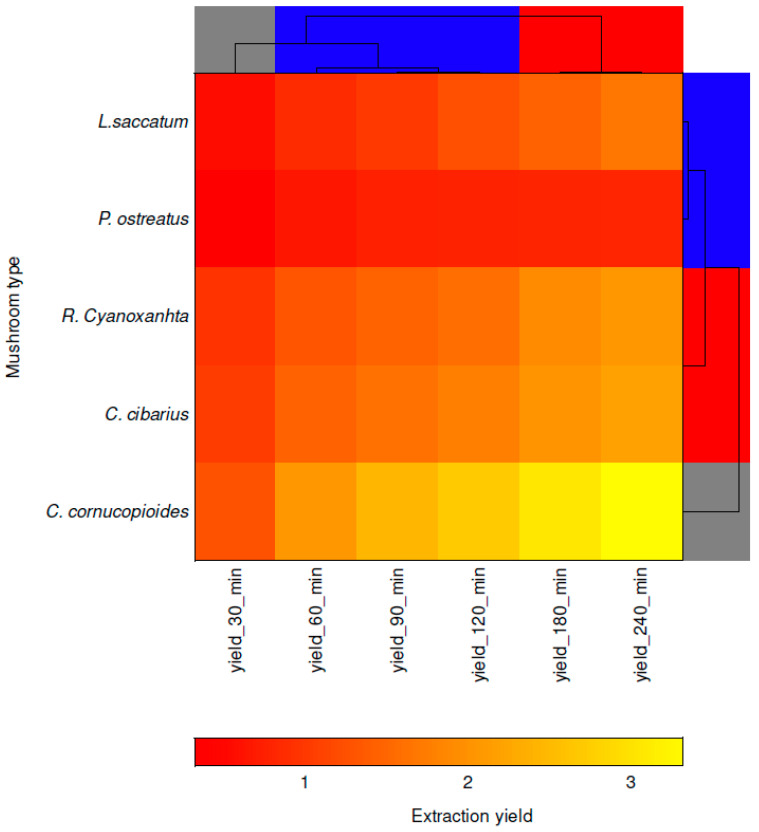
Clustered heat map (double dendrogram) of the extraction yield results.

A detailed explanation regarding the individual variables’ influence on grouping in the clusters is revealed by the clustered heat map in Figure 2. The presented double dendrogram indicates that the extraction yield results in 180 and 240 min (placed in the red horizontal cluster) are more similar than in the case of extraction yield results in 60, 90 and 120 min (placed in the blue horizontal cluster) for all mushroom types. The colour shades point out that four mushroom types (*L. saccatum* and *P. ostreatus* in one cluster, and *C. cibarius* and *R. Cyanoxantha* in the other one) have lower extraction yield values in all sampling times, while *C. cornucopioides* has the highest extraction yield values in all sampling times (30–240 min). Considering the grouping of the mushrooms, it can be seen that sample *C. cornucopioides* is out of the cluster. Additionally, the results of the conducted PCA and HCA analyses were confirmed by k-means clustering analysis. K-means divided the data set into three groups: (1) *C. cibarius* and *R.Cyanoxantha*, (2) *C. cornucopioides* and (3) *L. saccatum* and *P. ostreatus*.

## 4. Discussion

The supercritical extraction of *L.saccatum*, *P. Ostreatus*, *C. cornucopioides*, *R. Cyanoxantha* and *C. cibarius* was tested at a pressure of 30 MPa for an extraction time of 4 h. Extraction at a pressure of 300 bar (30 MPa) also proved to be the most effective in the study conducted by Mishra et al. [35], which was focused on *Cordyceps sinensis* and showed that the highest yield was obtained at pressure conditions of 300 bar and a temperature of 60 °C, which was superior to the yields obtained at 350 bar. Using Sc-CO_2_ extraction, Weber et al. [36] isolated high-value compounds from the mushroom *Agaricus bisporus*. They compared this very attractive method with conventional organic solvent extraction in order to compare both the yield and the chemical profile, as well as the bioactivity of the obtained extracts. During Sc-CO_2_ extraction, the pressure varied (20 and 30 MPa) while the temperature was constant (40 °C). As a result of the test, the higher efficiency of Sc-CO_2_ extraction compared to the conventional type, and also the higher efficiency of Sc-CO_2_ extraction at a higher pressure, was confirmed. The yield obtained during conventional extraction was 0.38 ± 0.01%, which was 2.47 fold less compared to Sc-CO_2_ extraction at 20 MPa (0.94 ± 0.13%), or 3.42 fold less compared to Sc-CO_2_ extraction at 30 MPa (1.30 ± 0.26%). In the same study, *A. bisporus* extracts obtained using Sc-CO_2_ extraction showed better performance in bioactive screening tests compared to the conventional ethanol extract. The same pressure was the most suitable for the Sc-CO_2_ extraction of bioactive compounds from *Boletus edulis* mushrooms, where it was determined that these mushrooms are rich in the content of linoleic acid [16]. On the other hand, Rodríguez-Seoane et al. [37], in their research using analysis of variances for Sc-CO_2_ *Pleurotus eryngii*, proved that the extraction is greatly affected by the applied extraction pressure, with the optimal conditions being achieved at 45 °C and 300 bar. In this case, the lowest yield was achieved at a pressure of 100 bar, which can be attributed to the decrease in carbon dioxide density with decreasing pressure, which consequently leads to a decrease in its dissolving power [38]. It can be observed that the effect of temperature is also very significant in the case of Sc-CO_2_ extraction. Thus, in the case of higher temperatures, there is an increase in solubility due to an increase in the vapour pressure of the dissolved substance, but at the same time, there is a decrease in the density of the solvent and a decrease in solubility, whereby the yield decreases [39]. According to all of the above, in our study, a temperature of 40 °C was chosen as a suitable working temperature at which the decomposition of thermolabile components is avoided, and a high yield of bioactive compounds is achieved.

Sc-CO_2_ extraction also showed a positive effect on the isolation of triterpenoids from mushrooms. From the medicinal mushroom *Inonotus oblikus* using Sc-CO_2_ extraction at three temperature-pressure combinations varying between 314 and 324 K (40–50 °C) and 281 and 350 bar, six triterpenoids were identified by GC–MS and quantified by GC–FID: ergosterol, lanosterol, β-sitosterol, stigmastanol, betulin and inotodiol [40]. On the other hand, Morales et al. [41] used this attractive method to isolate from *Lentinula edodes* fractions containing up to 18% (*v*/*v*) ergosterol and other ergosterol derivatives that could be further processed to induce partial transformation of this provitamin D_2_ into vitamin D_2_ by UV–light irradiation. Mishra et al. [35] obtained three different high-quality extracts by varying the conditions during the Sc-CO_2_ extraction of the medicinal mushroom *Cordyceps sinensis*. Thanks to the rich chemical composition (flavonoids, nucleobases and volatile organic compounds), the obtained extracts showed a wide range of bioactivity (antioxidant, antibacterial and hypoxia protective effects), which is why they can be effectively used for the development of mycotherapeutics, especially for recovery from hypobaric hypoxia.

From the data given in Table 2, it can be seen that the dominant compounds identified in the lipid extracts of all examined edible mushrooms are fatty acids, namely oleic + linoleic acid (47.848–64.796%) and palmitic acid (6.736–15.613%), followed by stearic acid (2.033–6.281%). In addition to the mentioned fatty acids, 10,13-octadecadiynoic acid (24.214%) was also identified in the lipid extract of *C. Cornucopioides*, which is a representative of the group of fatty acids that can be obtained from natural sources such as mushrooms, and the presence of triple bonds gives this molecule specific chemical properties such as the ability to polymerise (forming larger, more complex molecules).

Also, in all analysed mushroom extracts obtained by applying Sc-CO_2_ extraction, important compounds—sterols—were extracted. Some components of mushrooms that have a steroid structure are considered to possess, among others, antidiabetic and anti-arteriosclerosis effects; reduce cholesterol levels; and be significant in the prevention of colon cancer [42]. Therefore, the extraction of these natural compounds is of great importance. Matila et al. [43] confirmed that the leading compound of the steroid structure in mushroom extracts is ergosterol, which is in agreement with the results obtained in this study, except in the case of *L.saccatum*, where 7,22-ergostadienol and 7,22-ergostadienone are present in higher concentrations. Ergosterol, as a prominent sterol compound of great importance, was identified in all obtained Sc-CO_2_ extracts of the mentioned mushroom species.

The highest content was identified in the extract of *P. Ostreatus* and was 2.412%, followed by *L.saccatum* (1.707%) and *R. Cyanoxantha* (1.319%). In the remaining two species, the content of this sterol was below 1%, whereby the lowest yield (0.318%) was achieved in *C. Cornucopioides*. According to the yield, it can be concluded that *P. Ostreatus* is the most important source of this provitamin. Its yield is even 7.58 times higher compared to the yield obtained with *C. Cornucopioides*.

Ergosterol has been proven to possess various biological activities, such as anti-inflammatory, anticancer and antimicrobial properties [44]. Also, Li et al. [45] showed in their study that ergosterol purified from the medicinal mushroom *Amauroderma rude* was able to suppress the viability of breast cancer cells by inducing apoptosis in vitro and increasing the expression of tumour suppressors in vivo. Also, this compound is of great importance because it can be converted into vitamin D_2_ after photolysis and thermal rearrangement [46]. Therefore, ergosterol-rich mushrooms are considered the only source of vitamin D in vegetarians [47]. In addition to ergosterol, anthraergostateraenol was detected in supercritical extracts of *P. Ostreatus* and *C. Cornucopioides*. In the case of *L.saccatum*, 7,22-ergostadienone and 7,22-ergostadienol were identified as the leading sterol compounds while, in the case of *C. Cibarius*, in addition to the leading ergosterol, ergosta-4,6,8(14),22-tetraen-3-one and 7,22-ergostadienon were detected.

Using GC–FID, it was confirmed that the extracts of the examined mushrooms contain high concentrations of oleic, linoleic, stearic and palmitic fatty acids. In the extracts of *L.saccatum*, *P. Ostreatus*, *C. Cornucopioides*, *R. Cyanoxantha* and *C. Cibarius*, the content of the mentioned acids was 90.06%, 93.79%, 92.14%, 84.88% and 60.04%, respectively. It can be seen that the content of the mentioned acids is the lowest in the case of *C. Cibarius* (60.04%), while the highest amount of dominant acids was identified in the extract of *C. Cornucopioides* (93.79%). 10,13-octadecadiynoic acid methyl ester was detected in greater quantities in the extracts of *C. Cornucopioides* (24.05%) and *C. Cibarius* (5.29%). Also, in the *C. Cibarius* extract, the isomer of stearic acid was identified in the amount of 22.23%.

Some more fatty acids were detected in the obtained extracts of the examined mushrooms but in a much smaller proportion, such as palmitoleic, lauric, myristic and lignoceric acids. In the supercritical extracts, no fatty acids with long chains or fatty acids with a sequence lower than C10 were detected.

Analysing the literature, we concluded that fatty acids are very common compounds in mushrooms. Sande et al. [48] singled out linoleic, oleic and linolenic acids as the most abundant fatty acids that can be found in mushrooms worldwide. Also, their study confirmed mushrooms as an important source of essential fatty acids in the human diet. The lipid composition of *Morchella esculenta* grown in Bulgaria showed that in the examined species, the main fatty acids were linoleic (57.3%), palmitic (17.5%) and, to a lesser extent, linoleic (7.4%) acid [49]. Also, fatty acids were identified as constituents of methanol extracts of *Macrolepiota procera* and *Armillaria mellea* from two countries, Morocco and Portugal, although in a small amount, which proved the influence of the solvent on the nature of the isolated compounds [50].

Further, Mazzutti et al. [39] identified hexadecanoic (palmitic) acid and linoleic acid as the major acids in the Sc-CO_2_ extract of *A. brasiliensis*, where the compounds were associated with the antimicrobial activities of the extract.

Some other scientists were engaged in analysing the fatty acid profile of mushrooms. Shao et al. [51] investigated the fatty acid composition of the mushroom *A. bisporus*. According to their study, the total content of saturated fatty acids in *A. bisporus* was from 22.1 to 26.5%. Palmitic acid was the most present with a share of 14%, followed by stearic acid with a share of about 4%. Fatty acids with longer chains (26:0) were also detected, while saturated fatty acids with chains smaller than 10C atoms were not found, which is in accordance with earlier scientific research. And in this species, the dominant unsaturated fatty acid was linoleic acid, with a share of 67 to 76%.

Based on the previously mentioned data, it can be concluded that fatty acids are important and quantitatively present in various types of mushrooms. Apart from being an important source of energy, fatty acids also have many other important activities. They represent key components of phospholipids in cell membranes, which play a very important role in many cellular functions, including immune responses and metabolism [52]. Linoleic acid is an essential acid whose consumption is of great importance for the human body. Together with arachidonic acid (which it can convert into), it belongs to the omega-6 fatty acids that have been shown to have protective properties against cancer [53]. Linoleic acid possesses a number of biological and physiological benefits, including anti-carcinogenic [54], anti-obesity [55] and anti-diabetic [56] effects, as well as properties that promote bone formation [57]. Other highly represented acids in the extracts obtained in this study were saturated palmitic (hexadecanoic) acid and monounsaturated oleic acid. Palmitic acid also possesses a wide range of pharmacological activities, such as anti-inflammatory, antiviral and analgesic, as well as the regulatory activity of the lipid metabolism [58,59]. As for oleic acid, one of its most characteristic effects is its antioxidant capacity because it can directly regulate the synthesis and activities of antioxidant enzymes [60]. Some of the other beneficial effects are hypocholesterolemic and antitumor effects [61].

## 5. Conclusions

Regardless of the type consumed, this work represents another confirmation of the use of mushrooms as an important source of fatty acids and sterols in the human diet, although their concentration varies significantly depending on the location where they grow, the applied extraction method and climatic conditions. Also, the obtained values suggest that the supercritical extracts can be used as an attractive option in the functional food market while, due to the specificity of the high-pressure extraction technology applied, material left after extraction can be applied as safe and quality material for further functional ingredient processing, e.g., mushroom proteins and peptide production. The chemometric method gained a comprehensive approach to experimental data analysis and interpretation.

## Figures and Tables

**Table 1:** Extraction yield obtained during modern supercritical extraction and traditional *Soxhlet* extraction.

Type of Extraction	Extraction Time [min]	Extraction Yield [%]				
		Sample				
		*L. saccatum*	*P. ostreatus*	*C. cornucopioides*	*R. Cyanoxantha*	*C. cibarius*
Supercritical extraction	30	0.554 ± 0.03 ^pq^	0.330 ± 0.10 ^q^	1.272 ± 0.01 ^j–n^	0.949 ± 0.21 ^m–p^	1.035 ± 0.11 ^l–o^
	60	0.870 ± 0.06 ^nop^	0.646 ± 0.08 ^opq^	2.077 ± 0.06 ^def^	1.309 ± 0.19 ^j–m^	1.443 ± 0.16 ^i–l^
	90	1.010 ± 0.14 ^mno^	0.747 ± 0.23 ^op^	2.443 ± 0.11 ^cd^	1.459 ± 0.04 ^ijk^	1.608 ± 0.08 ^h–k^
	120	1.245 ± 0.27 ^k–n^	0.769 ± 0.71 ^op^	2.694 ± 0.03 ^bc^	1.582 ± 0.07 ^h–k^	1.775 ± 0.03 ^e–i^
	180	1.450 ± 0.16 ^ijk^	0.792 ± 0.85 ^op^	3.055± 0.05 ^ab^	1.915 ± 0.12 ^e–h^	2.028 ± 0.56 ^efg^
	240	1.664 ± 0.25 ^g–j^	0.807 ± 0.61 ^op^	3.316 ± 0.05 ^a^	2.061 ± 0.02 ^d–g^	2.180 ± 0.48 ^de^
Soxhlet extraction	480	1.75 ± 0.20 ^f–i^	0.83 ± 0.17 ^op^	3.38 ± 0.06 ^a^	2.11 ± 0.11 ^def^	2.47 ± 0.33 ^cd^

Different letters within a column indicate a significant difference between samples at *p* < 0.05.

**Table 2:** Compounds identified by GC–MS.

No.	Compound	Rt	*L. saccatum* [%]	*P. ostreatus* [%]	*C. cornucopioides* [%]	*R. Cyanoxantha* [%]	*C. cibarius* [%]
1.	3-methyl-butanoic acid	3.866	traces	-	-	traces	-
2.	hexanoic acid (caproic acid)	7.300	traces	-	-	-	0.338
3.	pantolactone	10.281	-	-	-	0.184	-
4.	phenylethyl alcohol	11.983	-	-	-	0.203	-
5.	succinimide	12.709	-	-	-	-	0.298
6.	2,4-dimethyl penthanal	14.322	-	1.087	-	-	-
7.	benzoic acid	14.738	traces	-	-	0.540	-
8.	6,6-dimethyl 2,4-heptanedione	15.565	-	0.322	-	-	-
9.	isothiocyanato cyclohexane	17.107	traces	-	-	traces	traces
10.	benzeneacetic acid	17.981	traces	-	-	1.077	traces
11.	2-methyl-3,5-dodecadiyne	18.608	-	-	0.154	-	-
12.	benzamide	21.575	0.237	-	-	-	-
13.	2-methyl crotonic acid	22.212	-	5.234	-	-	-
14.	2-methyl-2-penten-1-ol	23.248	-	1.139	-	-	-
15.	decanoic acid (capric acid)	23.307	-	-	-	0.164	-
16.	2-methyl-1-penten-3-ol	24.232	-	0.213	-	-	-
17.	cinnamic acid	25.827	traces	-	-	0.160	-
18.	5-pentyl resorcinol	29.886	-	0.332	-	-	-
19.	dodecanoic acid (lauric acid)	31.555	0.188	-	-	0.375	0.200
20.	tetradecanoic acid (myristic acid)	39.142	0.253	-	-	0.349	-
21.	pentadecanoic acid	42.771	1.323	1.012	0.479	0.487	0.298
22.	palmitoleic acid	45.801	-	-	-	3.517	-
23.	palmitic acid	46.816	8.398	6.736	12.259	8.615	15.613
24.	oleic acid + linoleic acid	53.066	64.796	57.544	47.848	61.891	55.581
25.	stearic acid	53.440	3.345	2.354	6.281	2.033	4.740
26.	10,13-octadecadiynoic acid	54.143	-	-	24.214	-	3.436
27.	6-oxo-octadecanoic acid	58.495	-	-	-	5.096	-
28.	methyl 10,13-octadecadiynoate	61.366	-	-	0.554	-	0.492
29.	α- glyceryl linoleate	74.650	-	0.321	-	-	-
30.	cis permethrin *	67.628	-	0.300	-	-	traces
31.	trans permethrin *	68.077	-	0.643	-	-	0.285
32.	anthraergostateraenol	78.660	-	0.297	0.103	-	-
33.	ergosterol	79.519	1.707	2.412	0.318	1.319	0.717
34.	7,22-ergostadienol	79.763	2.162	-	-	-	-
35.	ergosta-4,6,8(14),22-tetraen-3-one	83.266	-	-	-	-	0.173
36.	7,22-ergostadienone	80.463	3.580	-	-	-	0.299

* insecticide.

**Table 3:** GC–FID data on fatty acid composition percentage of total fatty acid methyl esters of supercritical wild edible mushroom extracts.

No.	Compound	Rt	*L. saccatum* [%]	*P. ostreatus* [%]	*C. cornucopioides* [%]	*R. Cyanoxantha * [%]	*C. cibarius* [%]
1.	12:0	6.00	0.30	-	0.13	0.73	-
2.	14:0	8.07	0.32	0.12	0.11	-	-
3.	15:0	9.36	1.55	0.31	0.43	0.89	-
4.	16:0	10.76	9.57	10.21	10.48	12.29	15.22
5.	16:1 t11	11.21	-	0.25	-	-	-
6.	16:1 c9	11.33	0.40	0.61	0.41	3.25	0.66
7.	17:0	11.60	0.77	-	-	-	0.80
8.	18:0	13.89	6.16	2.96	5.76	2.99	4.51
9.	18:1 t9 ^a^	14.20	-	0.74	-	-	-
10.	18:1 c9	14.36	6.64	40.25	26.44	27.99	9.80
11.	18:1 ^b^	14.54	1.22	1.50	0.36	1.93	22.23
12.	18:2 c10c13	15.09	-	-	0.34	-	-
13.	18:2 c9c12 ^c^	15.24	67.69	40.37	25.41	41.61	34.61
14.	18:3 c6c9c12	16.00	-	0.27	-	-	-
15.	20:0	17.20	-	-	0.11	-	-
16.	20:1 c11	17.70	-	0.31	0.25	-	-
17.	A ^d^	18.78	-	-	24.05	-	5.29
18.	22:0	20.29	0.36	0.13	-	-	-
19.	23:0	21.83	-	-	-	0.66	-
20.	24:0	23.33	-	-	0.18	1.65	4.47
21.	B ^e^	24.44	-	-	-	3.33	-

^a^ Trans-9-octadecanoic acid methyl ester; ^b^ exact structure of the isomer could not be determined; ^c^ Cis,cis-9,12-octadecadienoic acid methyl ester; ^d^ 10,13-octadecadiynoic acid methyl ester; ^e^ 6-oxo octadecanoic acid methyl ester.

## Data Availability

The original contributions presented in this study are included in the article. Further inquiries can be directed to the corresponding author.
